# The structural characterization of a glucosylglycerate hydrolase provides insights into the molecular mechanism of mycobacterial recovery from nitrogen starvation

**DOI:** 10.1107/S2052252519005372

**Published:** 2019-05-08

**Authors:** Tatiana Barros Cereija, Susana Alarico, Eva C. Lourenço, José António Manso, M. Rita Ventura, Nuno Empadinhas, Sandra Macedo-Ribeiro, Pedro José Barbosa Pereira

**Affiliations:** a IBMC – Instituto de Biologia Molecular e Celular, Universidade do Porto, Porto, Portugal; b Instituto de Investigação e Inovação em Saúde, Universidade do Porto, Porto, Portugal; c CNC – Centro de Neurociências e Biologia Celular, Universidade de Coimbra, Coimbra, Portugal; d IIIUC – Instituto de Investigação Interdisciplinar, Universidade de Coimbra, Coimbra, Portugal; e ITQB – Instituto de Tecnologia Química e Biológica António Xavier, Universidade Nova de Lisboa, Oeiras, Portugal

**Keywords:** *Mh*GgH, GH63, glycoside hydrolase, *Mycolicibacterium hassiacum*, protein structure, molecular recognition, X-ray crystallography, enzyme mechanism, solution scattering

## Abstract

Bacteria are challenged to adapt to environmental variations in order to survive. Under nutritional stress, several bacteria are able to slow down their metabolism into a nonreplicating state and wait for favourable conditions. It is almost universal that bacteria accumulate carbon stores to survive during this nonreplicating state and to fuel rapid proliferation when the growth-limiting stress disappears. Mycobacteria are exceedingly successful in their ability to become dormant under harsh circumstances and to be able to resume growth when conditions are favourable. Rapidly growing mycobacteria accumulate glucosylglycerate under nitrogen-limiting conditions and quickly mobilize it when nitrogen availability is restored. The depletion of intracellular glucosyl­glycerate levels in *Mycolicibacterium hassiacum* (basonym *Mycobacterium hassiacum*) was associated with the up-regulation of the gene coding for glucosylglycerate hydrolase (GgH), an enzyme that is able to hydrolyse glucosylglycerate to glycerate and glucose, a source of readily available energy. Highly conserved among unrelated phyla, GgH is likely to be involved in bacterial reactivation following nitrogen starvation, which in addition to other factors driving mycobacterial recovery may also provide an opportunity for therapeutic intervention, especially in the serious infections caused by some emerging opportunistic pathogens of this group, such as *Mycobacteroides abscessus* (basonym *Mycobacterium abscessus*). Using a combination of biochemical methods and hybrid structural approaches, the oligomeric organization of *M. hassiacum* GgH was determined and molecular determinants of its substrate binding and specificity were unveiled.

## Abstract 2

Glucosylglycerate accumulates to high levels in nitrogen-starved mycobacteria. Here, a thorough structural and biochemical characterization unveils the molecular determinants of the substrate specificity and catalytic mechanism of a mycobacterial glucosylglycerate hydrolase, a highly conserved enzyme involved in the fast recovery of rapidly growing mycobacteria from nitrogen starvation.

## 1. Introduction   

In a changing environment, the basic requirements for bacterial growth are not always available. In order to accomplish one single goal, survival, bacteria have evolved different strategies (Rittershaus *et al.*, 2013 ▸). When exposed to a growth-limiting stress, such as desiccation, temperature and pH variations, oxidative stress, hypoxia, antibiotics or nutrient limitation, bacterial populations balance between cell death and decreased growth rates (Finkel, 2006 ▸; Lipworth *et al.*, 2016 ▸; Eoh *et al.*, 2017 ▸). Some of the surviving cells can slow down or suspend their growth to a viable nonreplicating state and persist for months or years (Lewis, 2007 ▸). This process, which is known as dormancy, allows a viable population size to be maintained during the period of stress (Jones & Lennon, 2010 ▸). Despite being genetically identical to replicating bacteria, dormant cells are more tolerant to external stress (Balázsi *et al.*, 2011 ▸; Eldar & Elowitz, 2010 ▸; Dhar & McKinney, 2007 ▸). The dormancy state requires several structural modifications to maintain cell viability. Dormant cells seem to display a more compact and stable chromosome (Summers *et al.*, 2012 ▸; Nair & Finkel, 2004 ▸), a lower transcription rate and more stable messenger RNA (Rustad *et al.*, 2013 ▸), a lower but steady level of ATP (Gengenbacher *et al.*, 2010 ▸; Rao *et al.*, 2008 ▸), increased peptidoglycan mass in the cell wall accompanied by changes in the number and type of cross-links (Lavollay *et al.*, 2008 ▸; Zhou & Cegelski, 2012 ▸), and an accumulation of carbon stores (Bourassa & Camilli, 2009 ▸). Although the type of carbon store varies, the main common goal seems to be guaranteeing a rapidly mobilizable energy source that is able to promote fast cell proliferation when the environmental conditions improve (Shi *et al.*, 2010 ▸), which is an advantage at this moment in outcompeting neighbouring organisms.

In mycobacteria, the dormant state seems to be associated with the accumulation of triacylglycerol (Daniel *et al.*, 2004 ▸) and wax esters (Sirakova *et al.*, 2012 ▸). However, it has been demonstrated that rapidly growing mycobacteria accumulate glucosylglycerate (GG) under severe nitrogen-limiting conditions (Alarico *et al.*, 2014 ▸; Behrends *et al.*, 2012 ▸), which are able to induce dormancy (Anuchin *et al.*, 2009 ▸; Shleeva *et al.*, 2004 ▸). *In vitro*, GG prevented loss of activity in a number of enzymes (Sawangwan *et al.*, 2010 ▸), suggesting that it is also likely to contribute to protein stability *in vivo* during a slowly growing or nonreplicating phase. In *Mycolicibacterium smegmatis* and *M. hassiacum*, intracellular GG accumulated during nitrogen starvation is quickly depleted when nitrogen availability is restored (Alarico *et al.*, 2014 ▸; Behrends *et al.*, 2012 ▸). In *M. hassiacum*, GG depletion was associated with the up-regulation of a gene (*ggH*) coding for a glucosylglycerate hydrolase (GgH; Alarico *et al.*, 2014 ▸). Data on the role of GgH in bacterial survival, dormancy and infection are still lacking. However, the high degree of conservation of GgH among rapidly growing mycobacteria, which based on comparative genomic analyses were recently included in four new genera and separated from the *Mycobacterium* genus, where only the major human pathogens remain (Gupta *et al.*, 2018 ▸), and in other unrelated phyla suggests that the ability to produce this hydrolase is an evolutionary advantage. Moreover, since *ggH* expression is up-regulated upon relief of the growth-limiting stress, GgH is likely to participate in the reactivation of growth by hydrolysing GG to glycerate and glucose (Alarico *et al.*, 2014 ▸), which can be quickly used for energy production and for the synthesis of structural molecules (Mendes *et al.*, 2012 ▸) that are necessary for rapid bacterial proliferation. While most mycobacteria are environmental saprophytes, some species have increasingly been detected in oligotrophic drinking-water distribution systems, to which mycobacteria adapt and where they survive for long periods, namely in showerhead biofilms, from which they may access and infect humans (Gebert *et al.*, 2018 ▸). Understanding the mechanisms that allow the reactivation of growth following long periods under nutrient limitation may therefore be important in trying to halt the reactivation process and prevent infection.

*M. hassiacum* GgH (*Mh*GgH) belongs to the large CAZy family GH63 of glycoside hydrolases (http://www.cazy.org). Only four of the more than 2000 assigned members of family GH63, displaying α-glucosidase (EC 3.2.1.20), α-1,3-glucosidase (EC 3.2.1.84), processing α-glucosidase (EC 3.2.1.106) and mannosylglycerate hydrolase (EC 3.2.1.170) activities, have been structurally characterized (Kurakata *et al.*, 2008 ▸; Barker & Rose, 2013 ▸; Miyazaki *et al.*, 2015 ▸). In order to unveil its molecular mechanism of action, a thorough structural and functional characterization of *Mh*GgH was performed, elucidating its quaternary architecture and providing an atomic detail view of the determinants of substrate specificity, thus providing new insights into a potentially crucial enzyme underlying the metabolic reactivation of rapidly growing mycobacteria following severe nutrient starvation and expanding the options for therapeutic intervention.

## 2. Experimental procedures   

### 2.1. Site-directed mutagenesis   

The point mutations in *M. hassiacum ggH* were obtained by site-directed mutagenesis using pETM11-*Mh*GgH (Cereija *et al.*, 2017 ▸) as a template and the primers 5′-CACATGTGGAGTTGGGCCGCCGCGTTC-3′ and 5′-GAACGCGGCGGCCCAACTCCACATGTG-3′ (producing pETM11-D43A), 5′-GAGTCCGGGATGGCCAACTCG-3′ and 5′-CGAGTTGGCCATCCCGGACTC-3′ (producing pETM11-D182A), and 5′-TCGTTCGCCGCGTACTACGAA-3′ and 5′-TTCGTAGTACGCGGCGAACGA-3′ (producing pETM11-E419A).

### 2.2. Expression and purification of *Mh*GgH variants   

*Escherichia coli* BL21 (DE3) cells transformed with the pET30a-*Mh*GgH (Alarico *et al.*, 2014 ▸), pETM11-*Mh*GgH (Cereija *et al.*, 2017 ▸), pETM11-D43A, pETM11-D182A or pETM11-E419A plasmids were used for protein expression as described previously (Cereija *et al.*, 2017 ▸; Alarico *et al.*, 2014 ▸). All proteins were purified using a combination of immobilized metal-affinity and size-exclusion chromatography (Cereija *et al.*, 2017 ▸). The affinity tag of those proteins obtained from pETM11-based constructs was removed by cleavage with TEV protease. The concentration of the purified proteins (in 20 m*M* Tris–HCl pH 8.0, 400 m*M* NaCl; storage buffer) was estimated by measuring their absorbance at 280 nm prior to flash-freezing in liquid nitrogen and storage at −80°C until needed.

### 2.3. Analytical size-exclusion chromatography   

Analytical size-exclusion chromatography was performed on a Superdex 200 Increase 5/150 GL column (GE Healthcare) pre-equilibrated with storage buffer. Blue dextran (2000 kDa), ferritin (440 kDa), aldolase (158 kDa), conalbumin (75 kDa), ovalbumin (43 kDa) and ribonuclease A (13.7 kDa) were used as standards for column calibration. The *K*_av_ versus log molecular weight was calculated using the equation *K*_av_ = (*V*_e_ − *V*_0_)/(*V*_t_ − *V*_0_), where *V*_e_ is the elution volume of the protein, *V*_0_ is the void volume of the column and *V*_t_ is the column bed volume.

### 2.4. Dynamic light-scattering analysis   

Dynamic light-scattering (DLS) analysis was performed on a Zetasizer Nano ZS DLS system (Malvern Instruments). Protein samples were centrifuged at 13 000*g* and 4°C for 20 min, loaded onto a ZEN2112 cuvette and three independent measurements were recorded at 20°C. All data were analysed using the *Zetasizer* software v.7.11 (Malvern Instruments).

### 2.5. Differential scanning fluorimetry   

The melting temperatures of the *Mh*GgH variants were determined using a thermal shift (Thermofluor) assay. Each protein sample (0.5 mg ml^−1^ final concentration) was centrifuged at 13 000*g* and 4°C for 15 min, mixed with 5× SYPRO Orange (Life Technologies) in storage buffer and loaded into white 96-well PCR plates (Bio-Rad) sealed with Optical Quality Sealing Tape (Bio-Rad). The plate was heated from 25 to 95°C in 0.5°C steps with 30 s hold time per step on an iCycler iQ5 Multicolor Real-Time PCR Detection System (Bio-Rad) and the fluorescence was followed using a Cy3 dye filter (545 nm excitation/585 nm emission). Each experiment was performed in triplicate. The melting curves were analysed using the *CFX Manager* software (Bio-Rad) and the melting temperature was determined as the inflection point of the melting curve.

### 2.6. Chemical syntheses of glucosylglycerate, mannosylglycerate and glucosylglycolate   

The chemical syntheses of glucosylglycerate [GG; 2-*O*-(α-d-glucopyranosyl)-d-glycerate], mannosylglycerate [MG; 2-*O*-(α-d-mannopyranosyl)-d-glycerate] and glucosylglycolate [GGlycolate; 2-(1-*O*-α-d-glucopyranosyl)acetic acid] were performed as described previously (Faria *et al.*, 2008 ▸; Lourenço *et al.*, 2009 ▸; Lourenço & Ventura, 2011 ▸).

### 2.7. Substrate specificity of *Mh*GgH   

The catalytic activity of *Mh*GgH was evaluated in a 50 µl reaction mixture consisting of 2.75 µ*M* enzyme, 20 m*M* GG, 25 m*M* sodium phosphate pH 6.0, 5 m*M* MgCl_2_, 100 m*M* KCl (standard reaction). The hydrolysis of GG was detected by thin-layer chromatography (TLC; Silica Gel 60, Merck) using a solvent system composed of chloroform/methanol/acetic acid/water [30:50:8:4(*v*:*v*:*v*:*v*)] and the products were stained as described previously (Alarico *et al.*, 2014 ▸). Enzyme specificity was also probed with 5 and 20 m*M* MG, GGlycolate, glucosylglycerol [GGlycerol; 2-*O*-(α-d-glucopyranosyl)-d-glycerol; Bitop] or α-1,4-mannobiose (Carbosynth) as potential substrates. All reactions were performed at 42 and 50°C for 1 h, 3 h and overnight. Control reactions without enzyme were also performed. The reaction products were analysed by TLC as described above for GG, except for those from reactions containing GGlycerol, for which the solvent system used was chloroform/methanol/25% ammonia [30:50:25(*v*:*v*:*v*)].

### 2.8. Biochemical analysis and kinetic parameters of *Mh*GgH   

The effect of temperature and pH on the catalytic activity of *Mh*GgH was evaluated by quantifying the glucose released upon hydrolysis of GG using the Glucose Oxidase Assay Kit (Sigma–Aldrich) as described previously (Alarico *et al.*, 2014 ▸). The temperature profile of *Mh*GgH was traced from 20 to 60°C using the standard reaction conditions (see Section 2.7). The effect of pH on the activity of *Mh*GgH was determined at 55°C using 20 m*M* sodium acetate (pH 4.0–5.5) or 20 m*M* sodium phosphate (pH 5.8–7.0) buffer.

Kinetic parameters for the *Mh*GgH-catalysed hydrolysis of GG and MG were determined by quantifying the release of glucose (as described above) or mannose (using the K-MANGL 01/05 assay kit; Megazyme), respectively. A constant enzyme concentration (2.75 µ*M*) was incubated with increasing concentrations of GG (0–35 m*M*) or MG (0–150 m*M*) in 20 m*M* sodium phosphate pH 6.0, 100 m*M* KCl, 5 m*M* MgCl_2_ at 50°C. The maximum velocity (*V*_max_) and half constant (*K*_0.5_) were calculated with *Prism* 5.0 (GraphPad Software) using the allosteric sigmoidal equation. Experiments using GG were performed in triplicate, while those using MG as substrate were performed in duplicate.

### 2.9. Catalytic activity of *Mh*GgH variants   

The catalytic activity of *Mh*GgH variants was evaluated in 50 µl reactions consisting of 2.75 µ*M* enzyme and 10 or 20 m*M* GG or MG in 25 m*M* buffer (sodium phosphate pH 6.0, sodium acetate pH 4.5 or 5.0, bis-Tris propane pH 7.0 or Tris–HCl pH 8.0) with 5 or 10 m*M* MgCl_2_ in the presence or absence of 100 m*M* KCl. Reactions were performed at 37, 42 and 50°C for 1 h and overnight. Reaction mixtures with and without wild-type *Mh*GgH were used as controls. The hydrolysis of GG was evaluated by TLC as described in Section 2.7.

### 2.10. Crystallization of *Mh*GgH variants   

Crystals of *Mh*GgH were obtained as described previously (Cereija *et al.*, 2017 ▸). *Mh*GgH crystals growing from less than 30% GOL_P4K (glycerol, PEG 4000) were transferred into a solution containing at least 30% precipitant prior to flash-cooling in liquid nitrogen. The D43A, D182A and E419A *Mh*GgH variants were crystallized in the same conditions, although wild-type *Mh*GgH macro-seeds were employed to promote crystal growth. Complexes of the *Mh*GgH D182A variant with GG, MG and GGlycolate were obtained by soaking the crystals in mother liquor supplemented with 100 m*M* ligand for 2 h (GG and MG) or 25 min (GGlycolate) before flash-cooling in liquid nitrogen. Complexes of the *Mh*GgH E419A variant with GG, MG, GGlycolate and GGlycerol were obtained by soaking the crystals in mother liquor supplemented with 100 m*M* ligand for 5 min (GG), 50 min (MG), 40 min (GGlycolate) or 10 min (GGlycerol). An additional crystallization condition was identified in-house at 293 K, yielding hexagonal crystals within a day from drops consisting of equal volumes (1 µl) of protein (9.5 mg ml^−1^ in storage buffer) and precipitant solution equilibrated against solution No. 22 (0.1 *M* ADA pH 6.5, 1.0 *M* ammonium sulfate) from the MembFac sparse-matrix crystallization screen (Hampton Research). These crystals were cryoprotected with Perfluoropolyether PFO-X175/08 (Hampton Research) prior to flash-cooling in liquid nitrogen.

### 2.11. Data collection and processing   

Diffraction data were collected from cryocooled (100 K) single crystals on beamlines ID23-2 (Flot *et al.*, 2010 ▸), ID29 (de Sanctis *et al.*, 2012 ▸), ID30A-1 (Bowler *et al.*, 2015 ▸; Svensson *et al.*, 2015 ▸), ID30A-3 (Theveneau *et al.*, 2013 ▸) and ID30B (Mueller-Dieckmann *et al.*, 2015 ▸) of the European Synchrotron Radiation Facility (ESRF), Grenoble, France and PROXIMA-2A of the French National Synchrotron Source (SOLEIL), Gif-sur-Yvette, France. All data sets were automatically processed using the *GrenADES* (*Grenoble Automatic Data procEssing System*) pipeline (Monaco *et al.*, 2013 ▸), except for those collected on the ID30A-3 and PROXIMA-2A beamlines, which were processed with *XDS* (Kabsch, 2010 ▸) and reduced with utilities from the *CCP*4 program suite (Winn *et al.*, 2011 ▸). X-ray diffraction data-collection and processing statistics are summarized in Table 1 ▸. The X-ray diffraction images have been deposited in the SBGrid Data Bank (Meyer *et al.*, 2016 ▸).

### 2.12. Structure determination, model building and refinement   

The structure of *Mh*GgH was solved by multi-wavelength anomalous diffraction as reported previously (Cereija *et al.*, 2017 ▸). The refined coordinates were used as a search model to solve the structures of all of the other *Mh*GgH variants and complexes by molecular replacement using *Phaser* (McCoy *et al.*, 2007 ▸). Alternating cycles of model building with *Coot* (Emsley *et al.*, 2010 ▸) and refinement with *PHENIX* (Adams *et al.*, 2010 ▸) were performed until model completion. All crystallographic software was supported by SBGrid (Morin *et al.*, 2013 ▸). Refined coordinates and structure factors were deposited in the Protein Data Bank (Berman *et al.*, 2000 ▸). Refinement statistics are summarized in Table 1 ▸.

### 2.13. Analysis of crystallographic structures   

The crystallographic models were superposed with *SUPERPOSE* (Krissinel & Henrick, 2004 ▸) and the secondary-structure elements were identified with *DSSP* (Kabsch & Sander, 1983 ▸; Touw *et al.*, 2015 ▸). The interface area between the monomers was determined using *PISA* (Krissinel & Henrick, 2007 ▸). The surface electrostatic potential was calculated with *APBS* (Baker *et al.*, 2001 ▸) using the AMBER force field (Cornell *et al.*, 1995 ▸). Figures depicting molecular models were created with *PyMOL* (Schrödinger).

### 2.14. Small-angle X-ray scattering measurements and analysis   

Small-angle X-ray scattering (SAXS) measurements were recorded on beamline BM29 (Pernot *et al.*, 2013 ▸) of the ESRF, Grenoble, France with radiation of 0.9919 Å wavelength using a PILATUS 1M detector (Dectris). The protein sample was loaded onto a Superdex 200 3.2/300 GL column (GE Healthcare) and eluted with storage buffer. Measurements (1 Hz data-collection rate) were performed on the column eluate at 4°C over a scattering-vector (*s* = 4πsinθ/λ) range of 0.033–4.933 nm^−1^. Data were processed and analysed with the *ATSAS* package (Petoukhov *et al.*, 2012 ▸; Franke *et al.*, 2017 ▸). A Guinier plot was calculated using *PRIMUS/qt* (Konarev *et al.*, 2003 ▸). The theoretical scattering curve from the crystallo­graphic model was fitted to the experimental scattering curve with *CRYSOL* (Svergun *et al.*, 1995 ▸).

## 3. Results   

### 3.1. Catalytic activity of *Mh*GgH   

**Figure 1:**
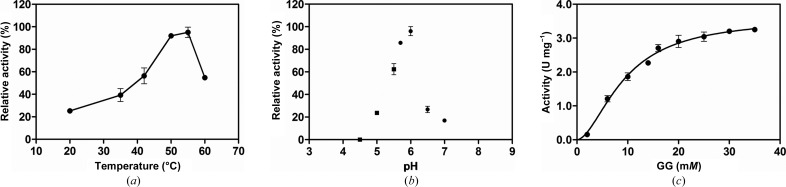
Biochemical characterization of *Mh*GgH. (*a*) Temperature profile of *Mh*GgH, highlighting its maximal activity at 50–55°C. (*b*) The effect of pH on the activity of *Mh*GgH assessed in 20 m*M* sodium acetate (squares) or 20 m*M* sodium phosphate (circles). (*c*) Kinetic curve using GG as the substrate. The sigmoidal shape of the experimental curve suggests the existence of a cooperative effect. Error bars correspond to standard deviations.

Recombinant *M. hassiacum* GgH (*Mh*GgH) containing only an additional Gly-Ala dipeptide at the N-terminus (Cereija *et al.*, 2017 ▸) was produced in *E. coli* and purified to homogeneity. *In vitro*, *Mh*GgH was able to hydrolyse both GG and MG, although with higher efficiency for the former. Under the conditions tested, this tag-less *Mh*GgH variant displayed maximum activity between 50 and 55°C, which was significantly higher than that reported for the C-terminally tagged variant (*Mh*GgH-His_6_, 42°C; Alarico *et al.*, 2014 ▸) and was in agreement with the optimal temperature of growth of *M. hassiacum* (50°C; Tiago *et al.*, 2012 ▸) [Fig. 1 ▸(*a*)].

At its optimal temperature, tag-less *Mh*GgH displayed maximum activity at pH 6.0 [Fig. 1 ▸(*b*)].

The kinetic parameters for *Mh*GgH were determined at 50°C. The experimental data were best fitted to an allosteric sigmoidal curve for both tag-less and tagged *Mh*GgH, with a Hill coefficient above 1, which suggests positive cooperativity [Fig. 1 ▸(*c*), Table 2 ▸]. The determined kinetic values for the hydrolysis of GG and MG by tag-less *Mh*GgH reflect an almost tenfold higher hydrolysis efficiency for GG (Table 2 ▸). Under the experimental conditions used it was not possible to obtain a complete kinetic curve for MG, resulting in a rough estimation of the value of *K*_0.5_ that nevertheless suggests higher affinity for GG.

### 3.2. Overall structure of *Mh*GgH   

Although both tagged and tag-less *Mh*GgH variants were used in crystallization experiments, only the tag-less variant yielded three-dimensional crystals. Orthorhombic crystals belonging to space group *P*2_1_2_1_2 that diffracted X-rays to beyond 1.7 Å resolution at a synchrotron source were obtained. Despite crystallizing in the same conditions, selenomethionine-substituted *Mh*GgH produced monoclinic crystals (space group *P*2_1_) that were used for structure solution by multi-wavelength anomalous diffraction at the *K* absorption edge of selenium as described previously (Cereija *et al.*, 2017 ▸). The orthorhombic crystals contained two *Mh*GgH molecules (here termed *A* and *B*) in the asymmetric unit, which were modelled from residues Pro2 to Gly446.

**Figure 2:**
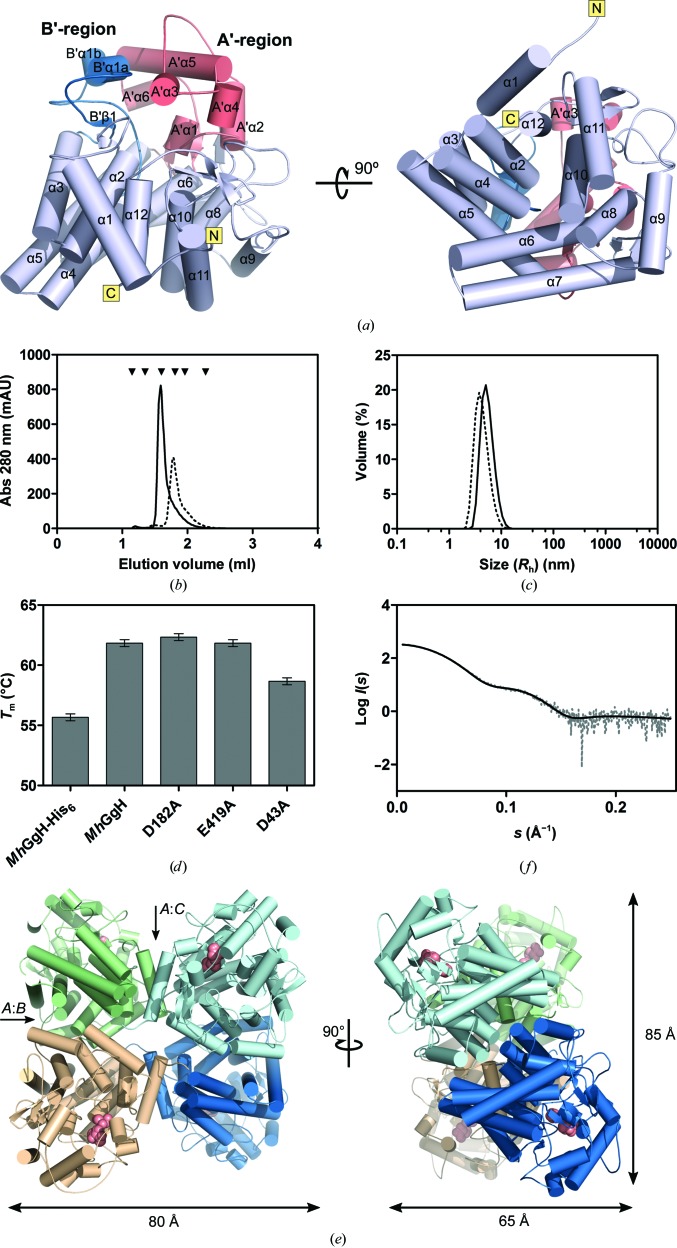
Structural and biophysical characterization of *Mh*GgH. (*a*) Cartoon representation of the overall structure of the *Mh*GgH monomer. The (α/α)_6_ domain is coloured mauve, and the A′- and B′-­regions are coloured salmon and blue, respectively. The N- and C-termini are indicated in yellow boxes. The views in the left and right panels are related by a 90° rotation around *x*. (*b*) Analytical size-exclusion chromatogram of tagged (dotted line) and tag-less (solid line) *Mh*GgH variants. The standards used for column calibration (see Section 2) are indicated as inverted black triangles. (*c*) Analysis of tagged (dotted line) and tag-less (solid line) *Mh*GgH variants by DLS. The tag-less variant displayed a larger hydro­dynamic radius (*R*_h_ = 7.34 nm) and a lower polydispersity index (PdI = 0.092) than the tagged *Mh*GgH variant (*R*_h_ = 5.75 nm; PdI = 0.201). (*d*) Melting temperatures of *Mh*GgH variants determined by differential scanning fluorimetry, highlighting the lower stability of the tagged *Mh*GgH variant. Error bars correspond to standard deviations. (*e*) Quaternary structure of *Mh*GgH. Monomers are coloured green (molecule *A*), wheat (molecule *B*), cyan (molecule *C*) and blue (molecule *D*). The *A*:*B* and *A*:*C* interfaces are indicated. The glycerol and serine molecules found in the active-site region are represented by salmon spheres. The approximate dimensions of the homotetramer are indicated. The views on the left and right are related by a 90° rotation around *y*. (*f*) Superposition of the experimental SAXS data (dotted grey line) and the theoretical SAXS curve calculated from the tetrameric crystallographic model of *Mh*GgH (solid black line).

The overall globular *Mh*GgH monomer [Fig. 2 ▸(*a*)] is composed of an (α/α)_6_-barrel domain that encompasses helices α2, α4, α6, α8, α10 and α12 in the inner layer and α1, α3, α5, α7, α9 and α11 in the outer layer, and a more flexible cap domain that constrains access to the active site of the enzyme and can in turn be divided into two subdomains termed the A′-region (residues 163–252) and the B′-region (residues 68–118). Five mobile loops are also noteworthy: loop A (between α1 and β1; residues 23–38), loop B (between B′β1 and B′α1a; residues 81–91), loop C (between A′α2b and A′α3; residues 193–205), loop D (between α9 and α10; residues 346–381) and loop E (between β6 and α12; residues 430–434) [Fig. 2 ▸(*a*), Supplementary Fig. S1].

*M. hassiacum* GgH displays 68% secondary-structure identity to the single structurally characterized mannosylglycerate hydro­lase *Thermus thermophilus* HB8 MgH (*Tt*8MgH; PDB entry 4wva; Miyazaki *et al.*, 2015 ▸), despite the much lower amino-acid sequence identity of 36% (calculated with *PDBeFold*; Krissinel & Henrick, 2004 ▸). To date, only three other MgH orthologues have been characterized biochemically: the MgH enzymes from *Selaginella moellendorffii* (Nobre *et al.*, 2013 ▸), *T. thermophilus* HB27 (99% amino-acid sequence identity to *Tt*8MgH) and *Rubrobacter radiotolerans* (Alarico *et al.*, 2013 ▸). Despite relatively low overall amino-acid sequence conservation, some regions are strictly conserved in *Mh*GgH and in all characterized MgH enzymes, including the putative catalytic Asp182 and Glu419 and most of the substrate-interacting residues (Tyr36, Trp40, Trp42, Asp43, Tyr88, Gln115, Gly180, Arg216, Tyr222, Tyr375 and Trp376) (Miyazaki *et al.*, 2015 ▸; Supplementary Fig. S2).

### 3.3. Quaternary structure of *Mh*GgH   

The apparent molecular weights of the *Mh*GgH variants in solution were evaluated by size-exclusion chromatography and DLS [Figs. 2 ▸(*b*) and 2 ▸(*c*)]. Both tagged and tag-less *Mh*GgH variants were analysed. While the *Mh*GgH-His_6_ variant displayed an apparent molecular weight of 92.6 kDa, corresponding to 1.8 times the expected mass of the monomer (50.9 kDa) and compatible with a dimeric arrangement, the apparent molecular weight of tag-less *Mh*GgH was 178.6 kDa, which is 3.5 times greater than the molecular weight of the monomer and is compatible with a trimeric or a tetrameric organization [Fig. 2 ▸(*b*)]. DLS analysis of the tagged and tag-less *Mh*GgH variants revealed an increase in the hydration radius (from 5.75 nm for tagged *Mh*GgH to 7.34 nm for the tag-less variant), which is in agreement with a higher oligomeric arrangement for tag-less *Mh*GgH, accompanied by a lower polydispersity index, which is indicative of higher homogeneity [Fig. 2 ▸(*c*)]. In agreement, tag-less *Mh*GgH displayed a significantly higher thermal stability (*T*_m_ = 62°C) than the tagged variant (*T*_m_ = 56°C) [Fig. 2 ▸(*d*)], explaining its higher optimal temperature of activity, and suggesting that the introduction of a hexahistidine tag at the C-terminus of *Mh*GgH affected protein stability by impairing quaternary-structure formation.

In the crystals, *Mh*GgH is arranged as a dimer of dimers with approximate dimensions of 85 × 80 × 65 Å [Fig. 2 ▸(*e*)]. The total surface area of each monomer is ∼17 300 Å^2^, of which ∼1600 Å^2^ is buried in intermonomer contacts. The largest interface area (∼900 Å^2^) occurs between molecules *A* and *C* (interface *A*:*C*) and molecules *B* and *D*, and involves 14 (*A*:*C*) or 12 (*B*:*D*) hydrogen bonds. With approximately half of the size (∼480 Å), the interface between dimers *A*:*B* and *C*:*D* is stabilized by four salt bridges. The smallest interface occurs between molecules *A* and *D* (and molecules *B* and *C*), with a buried surface of ∼260 Å^2^ and a single hydrogen bond (Supplementary Table S1). The C-terminus of each *Mh*GgH monomer is in the close vicinity of the *A*:*C* (or *B*:*D*) interface and the addition of the C-terminal affinity tag is likely to disrupt dimer–dimer association and impact the quaternary organization of the enzyme, which is in line with the observed lower maximum temperature of activity and decreased stability of the *Mh*GgH-His_6_ variant.

The oligomeric arrangement of *Mh*GgH in solution was also assessed by small-angle X-ray scattering (SAXS). The SAXS data are compatible with a tetrameric arrangement of the enzyme, and superposition of the experimental SAXS curve with that calculated from the crystallographic tetrameric model of *Mh*GgH reveals good agreement, further supporting that the crystallographic oligomer represents the quaternary architecture of the enzyme in solution [Fig. 2 ▸(*f*)].

### 3.4. Open and closed: mobility as an essential feature for substrate binding and hydrolysis   

**Figure 3:**
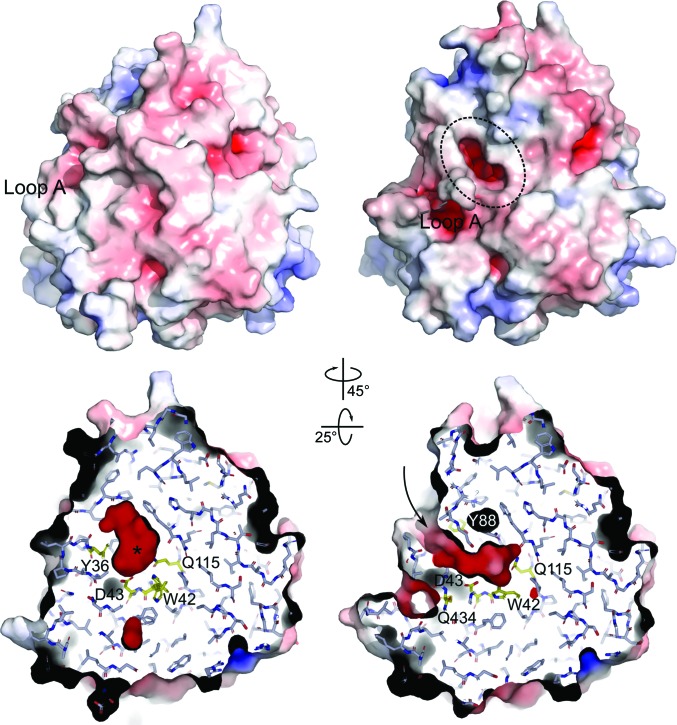
Closed and open conformations of *Mh*GgH. Solid-surface representation coloured according to electrostatic potential [contoured from −8 *kT*/e (red) to 8 *kT*/e (blue)] (upper panel) and cross-section (lower panel) of *Mh*GgH in closed (left) and open (right) conformations. In the closed state (left), the active-site cavity (marked with an asterisk) becomes inaccessible to the solvent. In the open state (right), an opening leading to an acidic cavity is observed (dashed ellipse; upper panel); a negatively charged tunnel (arrow) connects the active-site cavity to the exterior of the molecule (lower panel). Substrate-binding residues are highlighted in yellow. The left and right poses in each panel are related by 25° and 45° rotation around *x* and *y*, respectively.

In the orthorhombic crystals, the *Mh*GgH molecules adopt a closed conformation concomitant with the presence of two ligands, a molecule of glycerol and a molecule of serine, which are components of the crystallization buffer, at the active site (*Mh*GgH–Ser–GOL). The glycerol molecule occupies subsite −1 and serine is found at subsite +1 of the active site, inducing a closed state of *Mh*GgH that renders them inaccessible to the solvent (Fig. 3 ▸). These ligands are stabilized mainly by polar contacts, and the putative catalytic residues, Asp182 and Glu419, are facing the lumen of the active-site cavity.

In an alternative crystallization condition (space group *P*6_2_22), two *Mh*GgH protomers are present in the asymmetric unit, corresponding to molecules *A* and *C* of the tetramer observed in the orthorhombic crystals, which were modelled from Pro5 (molecule *A*) or Ala0 (molecule *C*) to Gly446. The active sites of both molecules contain only solvent (apo *Mh*GgH) and adopt an open conformation (Fig. 3 ▸). In the open conformation, the active site is accessible to the exterior through a negatively charged tunnel lined by the side chains of Trp40, Asp43, Tyr88, Gln115, Asp212, Ser214, Gln215, Met432, Gln433 and the carbonyl groups of Trp177 and Gly180 (Fig. 3 ▸). In contrast to the closed conformation, the putative catalytic residues point away from the active-site cavity: Asp182 is stabilized by polar contacts with the side chains of Tyr191 and Tyr225 and with the carbonyl group of Arg216 through a water molecule, whereas the side chain of Glu419 is hydrogen-bonded to the side chain of Ser435 and the amide N atoms of Met432 and Thr437 (Supplementary Fig. S3).

The two structures of *Mh*GgH, apo *Mh*GgH and *Mh*GgH–Ser–GOL, corresponding to its open and closed conformations, reveal the structural modifications that occur upon substrate binding. The active site of *Mh*GgH is surrounded by mobile loops that disclose the active site, exposing a polar surface for substrate binding. Indeed, several residues involved in substrate binding are present in these loops, including Tyr36 (loop A), His78, Tyr88 (loop B), Tyr375, Trp376 (loop D) and Gln434 (loop E). Access to the active site is additionally restricted by a flexible cap that also contains important residues for substrate binding (Gly180, Arg216 and Tyr222), as well as the putative catalytic residue Asp182. Substrate binding contributes to the formation of an additional helix in this cap (residues 206–209) that is absent in the open conformation (Supplementary Fig. S4).

A particularly significant modification is observed in the segment Arg21–Ala31 (loop A). In the closed conformation, this loop interacts with the segment Ser431–Ser435 (with polar interactions between the side chain of Asn23 and Gln433 and Ser435) that contains the substrate-interacting residue Gln434, which faces the active site and binds to the substrate. Moreover, upon substrate binding loop A is stabilized by polar contacts with the substrate via Tyr36 and with helices α3 and α12. In the open conformation, Gln433 and Gln434 move outwards (with displacements of their side chains of ∼11 and ∼8 Å, respectively), leading to a concerted rearrangement of loop E and loop A. Loop A (Arg21–Ala31) becomes mostly disordered, with difficult-to-interpret density that only allowed the modelling of two conformations for the segment Leu20–Leu25. In this short stretch, the contribution of Asp24-mediated interactions (with Arg21 or Arg58 of the neighbouring molecule) appears to be central to the overall conformation of this region. Moreover, given their spatial proximity, the conformation adopted by one subunit determines the position of the equivalent region of the neighbouring molecule, potentially impacting enzyme activity, which is in good agreement with the cooperative behaviour observed in the kinetics experiments [Fig. 1 ▸(*c*)].

### 3.5. Binding of substrates and substrate analogues to *Mh*GgH   

#### 3.5.1. Inactive variants of *Mh*GgH   

In order to understand the molecular determinants of substrate binding and specificity, *Mh*GgH was also crystallized in the presence of its substrates (GG and MG), substrate analogues (GGlycerol and GGlycolate) and reaction products (glucose, mannose and glycerate), and wild-type crystals were soaked in buffers containing these compounds. However, none of these approaches yielded crystals of the intended complexes.

To avoid substrate hydrolysis during crystallization or soaking, three catalytically inactive variants of *Mh*GgH were produced. The sequence variations were identified by homology to other characterized MgH enzymes (Supplementary Fig. S2) and analysis of the *Mh*GgH–Ser–GOL ternary-complex structure. Two putative catalytic residues (Asp182 and Glu419) and one substrate-interacting residue (Asp43) were identified and replaced by alanine to produce the D43A, D182A and E419A variants. The thermal stability of the D182A and E419A variants was comparable to that of wild-type *Mh*GgH, while that of the D43A variant was slightly lowered (3°C) [Fig. 2 ▸(*d*)]. None of the three variants displayed a detectable catalytic activity towards GG or MG (Supplementary Fig. S5).

#### 3.5.2. Inactive variants in complex with substrates   

**Figure 4:**
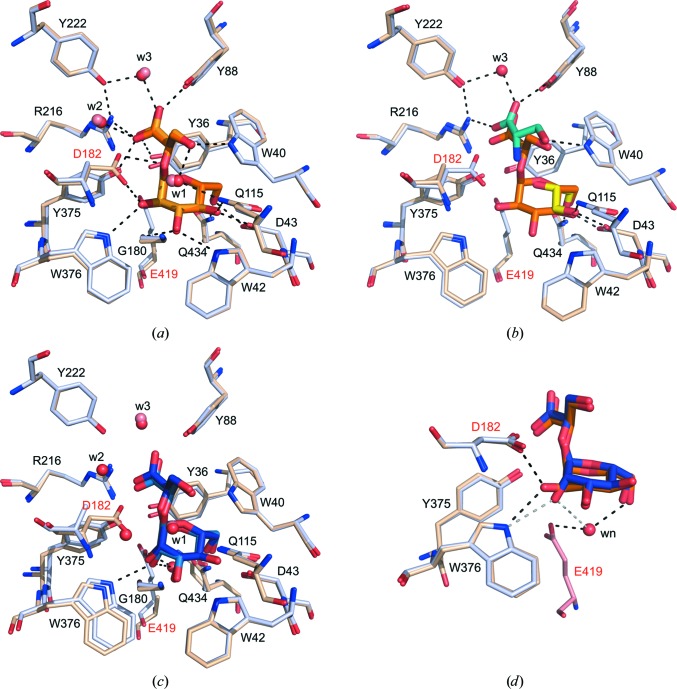
The active site of *Mh*GgH variants in complex with substrates. (*a*) Superposition of the active-site region of *Mh*GgH D182A and E419A variants in complex with GG. The position of GG (dark or light orange for the D182A or E419A variants, respectively) in the active site of the D182A (light blue) and E419A (wheat) variants is stabilized mainly by direct hydrogen bonds or by water (w)-mediated contacts (dashed lines) with the labelled residues. Water molecules in the D182A and E419A variants are represented by red and salmon spheres, respectively. The catalytic residues (Asp182 and Glu419) are highlighted in red. (*b*) Superposition of the active-site region of the D182A–GG complex (light blue with the ligand in orange) with that of the *Mh*GgH–Ser–GOL ternary complex (wheat). Hydrogen bonds between serine (cyan) or glycerol (yellow) and the residues of the active site are represented by dashed lines. (*c*) Superposition of the active-site regions of the *Mh*GgH D182A and E419A variants in complex with MG. The hydrogen-bonding network stabilizing MG at the active site is similar to that observed for GG [interacting residues are shown as in (*a*)]. The newly established contacts are represented by dashed lines. Water molecules (w) are coloured as in (*a*). (*d*) Superposition of the active-site region of the E419A variant of *Mh*GgH in complex with GG and MG and of the D182A variant in complex with MG (Glu419 in salmon). The hydrogen bonds between *Mh*GgH and GG (orange) or MG (blue) are represented by black or grey dashed lines, respectively. The nucleophilic water (wn) is also indicated.

Binary complexes of *Mh*GgH with GG or MG were obtained by soaking orthorhombic crystals of the inactive variants in mother liquor containing these compounds. There is residual electron density compatible with presence of the substrates in the structures of the D182A and E419A variants, but not in that of the D43A variant, independent of the soaking time, highlighting the contribution of Asp43 to substrate binding. For the D182A and E419A *Mh*GgH variants, the glucose/mannose moiety of each substrate occupies subsite −1 and the glycerate moiety occupies subsite +1 of the active site [Figs. 4 ▸(*a*) and 4 ▸(*c*)].

The active sites of *Mh*GgH in the D182A–GG and E419A–GG complexes are virtually identical (r.m.s.d. of 0.24 Å for 14 C^α^ pairs). The glucose moiety adopts a ^4^ *C*_1_ chair conformation with an α-anomeric configuration stabilized mainly by hydrogen bonds to the side chains of residues Trp42, Asp43, Gln115, Asp182, Tyr375, Trp376 and Gln434 and to the carbonyl group of Gly180, as well as by a water-mediated contact with Glu419 [Fig. 4 ▸(*a*)]. The glucose moiety is also oriented by hydrophobic contacts with Tyr36, Trp40, Trp376, Trp381 and Trp436. The glycerate moiety of the substrate is stabilized by polar contacts with Tyr36 (elongated in the D182A variant), Trp40, Tyr88, Arg216 and Tyr375. Water-mediated contacts with Phe89, Gln115, Trp177, Asp182 and Tyr222 also contribute to substrate binding, as do hydrophobic contacts with Trp177. One (D182A) or two (E419A) solvent molecules occupy the space and mimic the interactions of the missing side chains.

The substrate-interacting residues in the complexes between *Mh*GgH variants and GG are organized in a very similar way to that of *Mh*GgH–Ser–GOL [r.m.s.d.s of 0.19 Å (D182A) and 0.22 Å (E419A) for 14 C^α^ pairs]. The serine and glycerol molecules present in the active site of the *Mh*GgH–Ser–GOL structure partially mimic the substrate, with serine superposing nicely with the glycerate moiety and with glycerol establishing contacts with Asp43, a substrate placeholder that stabilizes the sugar moiety [Fig. 4 ▸(*b*)]. Taken together, these results suggest that the *Mh*GgH–Ser–GOL and variant–GG complexes are likely to reflect the substrate-binding mode of GG to wild-type *Mh*GgH.

The active site of both variants is also very similar when MG is bound (r.m.s.d. of 0.47 Å for 14 aligned C^α^ atoms) [Fig. 4 ▸(*c*)]. A similar hydrogen-bonding network stabilizes both substrates, with the most significant difference being the hydroxyl group at position C2 of the glucose/mannose moiety [Fig. 4 ▸(*d*)]. While the substrate O15 is hydrogen-bonded to Asp182 and Trp376 in the E419A–GG complex, in the E419A–MG complex only an elongated (3.5 Å) hydrogen bond to Trp376 is preserved, together with a novel water-mediated contact with Gln434.

#### 3.5.3. Mechanism of reaction   

At the active site of *Mh*GgH, the glycosidic O atom of both substrates is within hydrogen-bonding distance of the catalytic residue Asp182, while Glu419 establishes a water-mediated contact with both ligands. The distance between the catalytic residues and the substrate, as well as the presence of a single water molecule mediating the Glu419–substrate interaction, suggest that *Mh*GgH hydrolyses GG and MG through a classic inverting mechanism (Supplementary Fig. S6), with Asp182, Glu419 and a water molecule acting as the acid, base and nucleophile, respectively. During hydrolysis, the negatively charged Glu419 is likely to activate the water molecule that performs a nucleophilic attack on the anomeric C atom, while Asp182 donates a proton to the leaving glycerate. Owing to the positions of Glu419 and the water molecule, inversion of the anomeric configuration of the glucose/mannose upon hydrolysis is expected, in agreement with that previously observed for the MgH enzymes from *R. radiotolerans* and *T. thermophilus* HB27 (Alarico *et al.*, 2013 ▸).

#### 3.5.4. Inactive variants in complex with substrate analogues   

*Mh*GgH was unable to hydrolyse GGlycerol and GGlycolate, despite their considerable similarity to GG (Supplementary Fig. S7). The structures of the E419A variant in complex with both compounds and that of the D182A variant in complex with GGlycolate were determined and help in understanding this behaviour. The most significant differences from the complexes with GG/MG are observed at subsite +1. In the E419A–GGlycerol complex the glycerol moiety of the ligand is stabilized by a direct polar contact with Trp40 and by water-mediated contacts with Tyr88, Gln115, Trp177, Asp182 and Tyr222.

In the case of GGlycolate, the glycolate moiety is found in a single position in the E419A variant but adopts two different conformations in the D182A variant. In the E419A variant, this portion of the substrate analogue is stabilized by direct contacts with Trp40 and Tyr88 and by water-mediated inter­actions with Gln115, Trp177, His78 and Asp182. In the D182A variant, one of the glycolate conformations is close to that found in the E419A variant (direct contacts with Trp40 and Tyr88 and water-mediated interactions with Gln115 and Trp177), while the second conformation of the ligand is stabilized by hydrogen bonds to Tyr88, Arg216 and Tyr375 and by solvent-mediated contacts with Phe89, Trp177 and Tyr222.

Both substrate analogues establish fewer interactions with the active-site region of *Mh*GgH than bona fide substrates, which certainly impacts on the affinity of the enzyme for these compounds. In particular, interactions with Tyr36, a key residue for active-site closure and organization, are always absent, while different subsets of interactions are observed for GGlycerol and GGlycolate. It is therefore clear that substrate binding in *Mh*GgH is a well coordinated and fine-tuned event involving the concerted movement of flexible loops and interactions with highly conserved residues. Any deviation from these strict interaction patterns will result in a decreased affinity for and highly reduced activity towards the compound, as observed for GGlycerol and GGlycolate.

## 4. Discussion   

Mycobacteria encompass a large number of species, from the well known pathogen *M. tuberculosis*, which is able to cause tuberculosis in humans and in animals and is still the leading cause of death from a single infectious agent worldwide (World Health Organization, 2018 ▸), to the ubiquitous and opportunistic *M. abscessus* and the environmental and thermophilic *M. hassiacum*, which were recently included in the newly created genera *Mycobacteroides* and *Mycolicibacterium*, respectively (Gupta *et al.*, 2018 ▸). Although most of the known mycobacteria are considered to be nonpathogenic, an increasing number of infections by opportunistic non­tuberculous mycobacteria (NTM) have been reported over the last decade, which is likely to be a result of improved imaging techniques and molecular-sequencing methods that facilitate their identification (Alcaide *et al.*, 2017 ▸). On the other hand, ineffective sanitary control of water-distribution systems, as well as a number of host susceptibility factors, including ageing populations and an increased incidence of chronic diseases, may also be contributing factors to the increased rate of NTM infections detected worldwide (López-Varela *et al.*, 2015 ▸). Nontuberculous mycobacteria display high resilience against stress conditions, including an intrinsically high resistance to disinfectants and antibiotics; for this reason, NTM infections are a considerable clinical challenge for which therapeutic solutions are scarce (Falkinham, 2010 ▸). No significant advances in the treatment of NTM infections in general have recently been achieved, and the lengthy and toxic therapeutic plans in current use are often ineffective, which reinforces the need for more active drug development (Nessar *et al.*, 2012 ▸).

Although scarce, there are reports pointing to the accumulation of GG by environmental mycobacteria during nitrogen-limiting growth (Behrends *et al.*, 2012 ▸; Alarico *et al.*, 2014 ▸), a condition that is able to induce dormancy (Shleeva *et al.*, 2004 ▸; Anuchin *et al.*, 2009 ▸). As a compatible solute, GG can be accumulated intracellularly to high concentrations, and is a potential source of carbon and energy (Nunes-Costa *et al.*, 2017 ▸). Indeed, accumulated GG is quickly depleted upon exposure to an assimilable source of nitrogen, potentially fuelling bacterial growth. A glucosylglycerate hydrolase (GgH) identified in *M. hassiacum* and found to be highly conserved among rapidly growing mycobacteria is likely to be responsible for the rapid mobilization of GG accumulated during nitrogen starvation by hydrolysing it to glucose and glycerate (Alarico *et al.*, 2014 ▸). A recombinant form of this enzyme containing a C-terminal hexahistidine tag has been characterized biochemically (Alarico *et al.*, 2014 ▸), but failed to form three-dimensional crystals suitable for structural studies. An alternative construct containing a cleavable N-terminal hexahistidine tag was recently generated (Cereija *et al.*, 2017 ▸) and removal of the affinity tag yielded an *Mh*GgH variant with an additional N-terminal Gly-Ala dipeptide, which readily crystallized in two different conditions and diffracted X-rays to 1.7 Å resolution. The crystallographic structure of *Mh*GgH revealed a homotetrameric architecture, which is compatible with the oligomeric organization of the enzyme in solution as assessed by SAXS. The C-terminus of *Mh*GgH was found to be involved in monomer–monomer association, which was likely to be impaired by the C-terminal placement of the affinity tag in the original construct, also explaining the lower stability of this variant.

The *Mh*GgH monomer displays an (α/α)_6_-barrel domain typical of glycoside hydrolase family 63 (GH63), to which *Mh*GgH belongs. While the active site of GH63 members acting on larger substrates is located in an open, solvent-accessible cleft, those of *Mh*GgH and of MgH from *T. thermophilus* are covered by a cap domain (subdivided into A′- and B′-regions) with constrained access through a narrow negatively charged tunnel. Upon substrate binding, the cap domain closes, establishing contacts necessary to stabilize and orient the small substrate and to prevent access of bulk solvent to the active site. The open and closed states of *Mh*GgH are determined by the well coordinated movement of several mobile loops that contain some of the substrate-interacting residues.

A kinetic study of *Mh*GgH revealed a cooperative effect between the units of the tetramer, which may result from intersubunit interactions mediated by the mobile loop A. Indeed, in the open conformation loop A regions from adjacent monomers interact, potentially impacting on the enzymatic activity. Substrate binding by one subunit leads to the stabilization of its loop A, which is likely to facilitate access to the active site of the neighbouring subunit.

*Mh*GgH was able to hydrolyse GG more efficiently than MG *in vitro*, in contrast to the similar efficiency for both substrates displayed by the MgH orthologues from *T. thermophilus* and *S. moellendorfii* (Nobre *et al.*, 2013 ▸; Alarico *et al.*, 2013 ▸). This behaviour of *Mh*GgH could be explained by its distinct binding affinities for the two compounds. The α-d-glucose and α-d-mannose moieties of GG and MG, respectively, differ in the orientation of the C2 hydroxyl group, which is equatorial in α-d-glucose and axial in α-d-mannose. As a consequence, the C2 hydroxyl group of the glucose moiety of GG establishes polar contacts with Asp182 and Trp376, while that of the mannose moiety of MG establishes a single contact with Trp376. The larger number of interactions between GG and *Mh*GgH are likely to translate into a higher affinity of binding and to explain the preference of the enzyme for this substrate.

The contribution of subsite +1 to substrate recognition was also evaluated using the substrate analogues GGlycerol and GGlycolate, which differ from GG in the aglycone moiety. *In vitro*, *Mh*GgH was unable to hydrolyse either compound. The structures of catalytically inactive variants of *Mh*GgH in complex with these substrate analogues were determined, revealing the important contribution of the reducing end of the substrate to active-site closure and the ensuing enzymatic processing.

Substrate binding to *Mh*GgH is a well coordinated event involving highly conserved residues and a complex network of polar contacts. During evolution, the active site of *Mh*GgH has been optimized to harbour specific substrates, such as GG. The presence of MG in *M. hassiacum* cells has not so far been reported. Assuming that *M. hassiacum* is unable to produce MG, the ability of *Mh*GgH to hydrolyse this compound may reflect a vestigial function from an ancestor enzyme. On the other hand, since cells are able to exchange molecules with the environment using different strategies, from passive diffusion to active transport, it is also possible that *M. hassiacum* possesses adequate machinery for scavenging MG released by other organisms from the environment through active export or cell death (Sampaio *et al.*, 2004 ▸) as a potential source of carbon and energy mobilized through hydrolysis by *Mh*GgH.

The antibiotics currently in use mainly counter DNA replication or RNA, protein or cell-wall synthesis, which are indispensable functions for cell growth (Kohanski *et al.*, 2010 ▸). Since these processes are almost suppressed in dormant cells, they are more likely to survive treatment with antibiotics. Indeed, dormant cells have been associated with post-treatment relapse and the development of genetic resistance (Levin & Rozen, 2006 ▸; Gomez & McKinney, 2004 ▸). As a likely intervenient in cell recovery upon nitrogen stress relief, GgH may be viewed as a potential target for the development of new more efficient antimycobacterial drugs. Its comprehensive structural characterization contributes to clarification of the molecular determinants of substrate binding and specificity and provides a detailed molecular scaffold for the rational design of specific inhibitors.

## 5. Related literature   

The following references are cited in the Supporting Information for this article: Bond & Schüttelkopf (2009 ▸) and Sievers *et al.* (2011 ▸).

## Supplementary Material

PDB reference: *Mh*GgH, apo, 6q5t

PDB reference: without serine, 5ohc

PDB reference: SeMet, 5ohz

PDB reference: *Mh*GgH–Ser–GOL, 5oi0

PDB reference: D182A–GG, 5oiw

PDB reference: D43A–Ser–GOL, 5oiv

PDB reference: E419A–GG, 5oju

PDB reference: D182A–MG, 5oj4

PDB reference: E419A–Ser–GOL, 5oie

PDB reference: D182A–Ser–GOL, 5oi1

PDB reference: E419A–MG, 5ojv

PDB reference: E419A–GGycerol, 5ont

PDB reference: D182A–GGlycolate, 5onz

PDB reference: E419A–GGlycolate, 5oo2

Supporting tables and figures. DOI: 10.1107/S2052252519005372/jt5034sup1.pdf

X-ray diffraction data from Mycobacterial glucosylglycerate hydrolase - Se-Met derivative, source of 5OHZ structure: https://doi.org/10.15785/SBGRID/465

X-ray diffraction data from Mycobacterial glucosylglycerate hydrolase, source of 5OI0 structure: https://doi.org/10.15785/SBGRID/467

X-ray diffraction data from Mycobacterial glucosylglycerate hydrolase, source of 5OHC structure: https://doi.org/10.15785/SBGRID/464

X-ray diffraction data from Mycobacterial glucosylglycerate hydrolase, source of 6Q5T structure: https://doi.org/10.15785/SBGRID/641

X-ray diffraction data from Mycobacterial glucosylglycerate hydrolase - D43A mutant, source of 5OIV structure: https://doi.org/10.15785/SBGRID/471

X-ray diffraction data from Mycobacterial glucosylglycerate hydrolase - D182A mutant, source of 5OI1 structure: https://doi.org/10.15785/SBGRID/468

X-ray diffraction data from Mycobacterial glucosylglycerate hydrolase - E419A mutant, source of 5OIE structure: https://doi.org/10.15785/SBGRID/470

X-ray diffraction data from Mycobacterial glucosylglycerate hydrolase - D182A mutant in complex with glucosylglycerate, source of 5OIW structure: https://doi.org/10.15785/SBGRID/472

X-ray diffraction data from Mycobacterial glucosylglycerate hydrolase - D182A mutant in complex with mannosylglycerate, source of 5OJ4 structure: https://doi.org/10.15785/SBGRID/473

X-ray diffraction data from Mycobacterial glucosylglycerate hydrolase - D182A mutant in complex with glucosylglycolate, source of 5ONZ structure: https://doi.org/10.15785/SBGRID/482

X-ray diffraction data from Mycobacterial glucosylglycerate hydrolase - E419A mutant in complex with glucosylglycerate, source of 5OJU structure: https://doi.org/10.15785/SBGRID/474

X-ray diffraction data from Mycobacterial glucosylglycerate hydrolase - E419A mutant in complex with mannosylglycerate, source of 5OJV structure: https://doi.org/10.15785/SBGRID/475

X-ray diffraction data from Mycobacterial glucosylglycerate hydrolase - E419A mutant in complex with glucosylglycolate, source of 5OO2 structure: https://doi.org/10.15785/SBGRID/483

X-ray diffraction data from Mycobacterial glucosylglycerate hydrolase - E419A mutant in complex with glucosylglycerol, source of 5ONT structure: https://doi.org/10.15785/SBGRID/481

## Figures and Tables

**Table:** Values in parentheses are for the outermost shell.

Crystal	SeMet *Mh*GgH	*Mh*GgH–Ser–GOL	*Mh*GgH without serine	Apo *Mh*GgH	D43A–Ser–GOL	D182A–Ser–GOL	E419A–Ser–GOL
Data collection							
Synchrotron-radiation facility	ESRF	ESRF	ESRF	ESRF	ESRF	ESRF	ESRF
Beamline	ID29	ID30B	ID30A-3	ID29	ID30A-1	ID30A-3	ID30A-1
Detector	PILATUS3 6M, Dectris	PILATUS 6M, Dectris	EIGER X 4M, Dectris	PILATUS3 6M, Dectris	PILATUS3 2M, Dectris	EIGER X 4M, Dectris	PILATUS3 2M, Dectris
Wavelength (Å)	0.97909	0.97265	0.96770	0.96863	0.96598	0.96770	0.96600
Reflections (measured/unique)	428683/151619	595791/135623	390265/84736	474316/66272	460425/114314	581967/119738	338529/73877
Space group	*P*2_1_	*P*2_1_2_1_2	*P*2_1_2_1_2	*P*6_2_22	*P*2_1_2_1_2	*P*2_1_2_1_2	*P*2_1_2_1_2
*a*, *b*, *c* (Å)	90.8, 86.1, 159.7	86.0, 158.8, 87.8	85.9, 159.3, 91.2	167.0, 167.0, 243.3	85.9, 159.1, 88.2	86.3, 158.1, 87.7	86.2, 159.4, 88.4
α, β, γ (°)	90.0, 93.0, 90.0	90.0, 90.0, 90.0	90.0, 90.0, 90.0	90.0, 90.0, 120.0	90.0, 90.0, 90.0	90.0, 90.0, 90.0	90.0, 90.0, 90.0
Resolution (Å)	57.4–2.04 (2.07–2.04)	48.6–1.68 (1.74–1.68)	40.3–2.00 (2.04–2.00)	49.9–2.54 (2.63–2.54)	48.7–1.78 (1.85–1.78)	42.2–1.75 (1.78–1.75)	48.8–2.07 (2.14–2.07)
*R*_merge_	0.105 (0.637)	0.047 (0.860)	0.084 (1.276)	0.109 (1.165)	0.057 (0.824)	0.070 (0.987)	0.094 (1.032)
〈*I*/σ(*I*)〉	7.6 (1.6)	16.4 (1.7)	12.1 (1.2)	11.9 (1.8)	13.0 (1.7)	11.6 (1.6)	10.0 (1.3)
Completeness (%)	96.5 (82.1)	99.0 (98.4)	99.6 (99.8)	99.8 (99.7)	98.9 (93.5)	98.8 (99.3)	98.9 (92.6)
Multiplicity	2.8 (2.8)	4.4 (4.5)	4.6 (4.8)	7.2 (7.3)	4.0 (4.0)	4.9 (5.0)	4.6 (4.0)
Refinement							
Resolution (Å)	57.4–2.04	48.6–1.68	40.3–2.00	49.9–2.54	45.4–1.78	40.0–1.75	48.8–2.07
*R*_work_/*R*_free_ (%)	19.9/24.5	14.6/17.2	16.7/21.0	16.6/20.4	14.9/17.9	14.6/17.2	16.7/20.8
No. of reflections							
Working set	255146	135565	84685	66183	114235	119673	73714
Test set	12657	6808	4330	3359	5735	5953	3718
Total No. of atoms	15955	8595	8115	7635	8365	8421	7767
Ligands at active site	SER, GOL	SER, GOL	GOL		SER, GOL	SER, GOL	SER, GOL
No. of water molecules	1289	838	705	379	761	776	420
Wilson *B* factor (Å^2^)	28.4	25.1	33.4	53.0	29.3	26.0	39.9
R.m.s. deviations							
Bond lengths (Å)	0.010	0.010	0.010	0.009	0.010	0.010	0.010
Bond angles (°)	1.099	1.031	0.993	1.058	1.009	1.019	1.004
Ramachandran plot							
Favoured (%)	95.6	96.8	96.7	96.0	97.0	97.4	96.3
Allowed (%)	4.3	3.2	3.3	3.6	3.0	2.6	3.6
Outliers (%)	0.1	0.0	0.0	0.4	0.0	0.0	0.1
Molecules in asymmetric unit	4	2	2	2	2	2	2
PDB code	5ohz	5oi0	5ohc	6q5t	5oiv	5oi1	5oie
SBGrid code	465	467	464	641	471	468	470

**Table:** 

Crystal	D182A–GG	D182A–MG	D182A–GGlycolate	E419A–GG	E419A–MG	E419A–GGlycolate	E419A–GGlycerol
Data collection							
Synchrotron-radiation facility	ESRF	ESRF	ESRF	ESRF	ESRF	SOLEIL	ESRF
Beamline	ID30A-1	ID30A-1	ID23-2	ID30A-1	ID30B	PROXIMA-2A	ID30A-3
Detector	PILATUS3 2M, Dectris	PILATUS3 2M, Dectris	PILATUS3 2M, Dectris	PILATUS3 2M, Dectris	PILATUS 6M, Dectris	EIGER X 9M, Dectris	EIGER X 4M, Dectris
Wavelength (Å)	0.96600	0.96600	0.87290	0.96599	0.97625	0.98011	0.96770
Reflections (measured/unique)	801298/133666	780685/117039	547439/91224	301215/64817	475955/74395	500266/75133	322372/76309
Space group	*P*2_1_2_1_2	*P*2_1_2_1_2	*P*2_1_2_1_2	*P*2_1_2_1_2	*P*2_1_2_1_2	*P*2_1_2_1_2	*P*2_1_2_1_2
*a*, *b*, *c* (Å)	85.3, 159.6, 91.0	85.3, 159.6, 91.2	86.2, 158.9, 88.0	86.1, 159.1, 88.4	87.8, 158.2, 87.6	86.9, 157.7, 87.6	86.9, 158.8, 87.7
α, β, γ (°)	90.0, 90.0, 90.0	90.0, 90.0, 90.0	90.0, 90.0, 90.0	90.0, 90.0, 90.0	90.0, 90.0, 90.0	90.0, 90.0, 90.0	90.0, 90.0, 90.0
Resolution (Å)	49.1–1.71 (1.77–1.71)	49.1–1.79 (1.85–1.79)	48.7–1.93 (2.00–1.93)	48.7–2.17 (2.25–2.17)	48.8–2.06 (2.13–2.06)	45.1–2.06 (2.10–2.06)	45.3–2.05 (2.09–2.05)
*R*_merge_	0.070 (1.081)	0.074 (1.052)	0.126 (1.227)	0.091 (0.790)	0.086 (0.979)	0.124 (1.410)	0.091 (0.901)
〈*I*/σ(*I*)〉	13.8 (1.8)	16.5 (1.7)	8.8 (1.5)	11.3 (1.8)	12.1 (1.5)	8.2 (1.3)	9.4 (1.6)
Completeness (%)	99.5 (99.0)	99.4 (95.9)	99.8 (98.7)	99.8 (99.5)	98.0 (89.3)	100.0 (100.0)	99.4 (100.0)
Multiplicity	6.0 (6.1)	6.7 (6.5)	6.0 (6.2)	4.6 (4.5)	6.4 (6.0)	6.7 (6.9)	4.2 (4.4)
Refinement							
Resolution (Å)	45.9–1.71	49.1–1.79	48.7–1.93	45.5–2.17	48.8–2.06	41.9–2.06	45.3–2.05
*R*_work_/*R*_free_ (%)	14.6/16.8	14.4/17.3	14.9/18.5	15.6/19.8	15.5/19.4	16.2/20.2	15.4/19.5
No. of reflections							
Working set	133615	116974	91157	64760	74327	75046	76246
Test set	6717	5861	4566	3281	3733	3625	3909
Total No. of atoms	8376	8421	8283	7817	7840	7868	7944
Ligands at active site	9WN	2M8	GOL, SER, 9YW	9WN	GOL, SER, 2M8	GOL, SER, 9YW	A0K
No. of water molecules	847	846	677	419	463	426	566
Wilson *B* factor (Å^2^)	24.9	25.6	28.3	35.7	39.5	39.7	33.7
R.m.s. deviations							
Bond lengths (Å)	0.010	0.010	0.010	0.009	0.009	0.010	0.010
Bond angles (°)	1.025	0.986	0.984	1.005	0.985	1.030	1.011
Ramachandran plot							
Favoured (%)	97.2	96.5	97.0	96.6	97.5	96.5	96.4
Allowed (%)	2.8	3.5	3.0	3.3	2.4	3.4	3.1
Outliers (%)	0.0	0.0	0.0	0.1	0.0	0.1	0.5
Molecules in asymmetric unit	2	2	2	2	2	2	2
PDB code	5oiw	5oj4	5onz	5oju	5ojv	5oo2	5ont
SBGrid code	472	473	482	474	475	483	481

**Table 2:** Kinetic parameters for hydrolysis of GG and MG Experimental data were analysed using the allosteric kinetic model. A lower affinity for MG is expected owing to the higher estimated *K*_0.5_ value.

Kinetic parameters	GG	MG
*V*_max_ (µmol min^−1^ per milligram of protein)	3.60 ± 0.18	3.09 ± 0.66
*K*_0.5_ (m*M*)	9.36 ± 0.69	84.18 ± 30.27
*h*	1.77 ± 0.20	1.29 ± 0.23
*R* ^2^	0.974	0.990
