# Supplementary material for: The structural characterization of a glucosylglycerate hydrolase provides insights into the molecular mechanism of mycobacterial recovery from nitrogen starvation
Source: IUCrJ. 2019 May 8;6(Pt 4):572–85. doi: 10.1107/S2052252519005372 (PMC6608630; doi:10.1107/S2052252519005372)
Supplement: Supplementary file 1 [file m-06-00572-sup1.pdf]

IUCrJ

Volume 6 (2019)

Supporting information for article:

The structural characterization of a glucosylglycerate hydrolase provides insights into the molecular mechanism of mycobacterial recovery from nitrogen starvation

Tatiana Barros Cereija, Susana Alarico, Eva C. Lourenço, José António Manso, M. Rita Ventura, Nuno Empadinhas, Sandra Macedo-Ribeiro and Pedro José Barbosa Pereira

Table S1 Analysis of the quaternary structure of *Mh*GgH. The total surface and total interface areas of each monomer (upper table) and the intermonomer area and interactions (lower table) were determined using *PISA* (Krissinel & Henrick, 2007).

Monomer	Total surface area (Å^2)	Total interface area (Å^2)
A	17378	1637
B	17218	1633
C	17381	1639
D	17216	1633
Average	17298	1636

Interface	Interface area (Å^2)	Interface interaction	
		Hydrogen bonds	Salt bridges
A:B	477	0	4
C:D	478	0	4
A:C	900	14	0
B:D	895	12	0
A:D	260	1	0
B:C	261	1	0

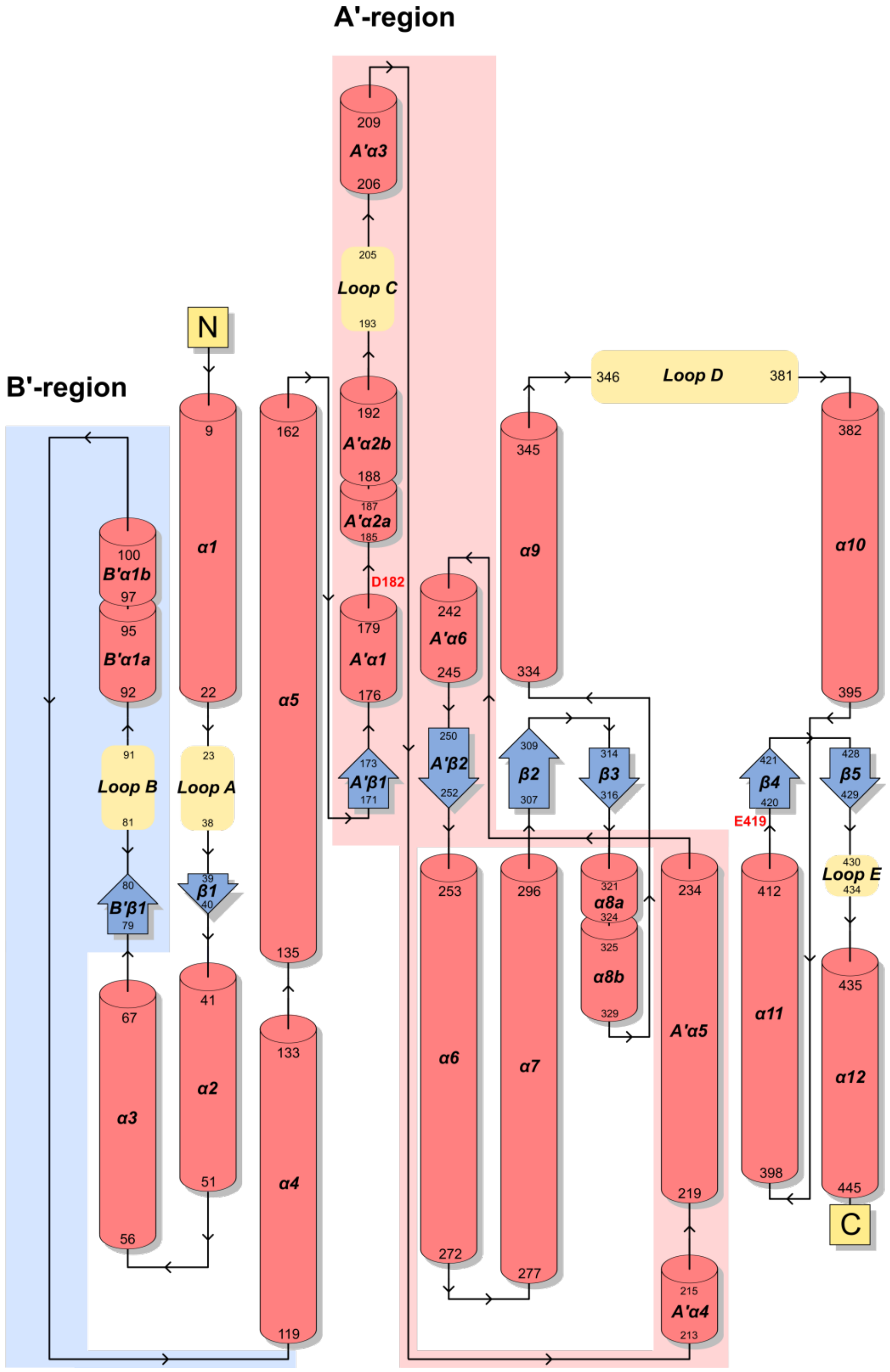

Figure S1 Topology diagram of *Mh*GgH. Helices (α1 to α12, A'α1 to A'α6, and B'α1), strands (β1 to β5, A'β1 to A'β2, and B'β1) and mobile loops (loop A to loop E) are represented as salmon cylinders, blue arrows and yellow quadrilaterals, respectively. Catalytic residues are labelled red. The A' and B'-regions are highlighted by pink and blue shapes, respectively.

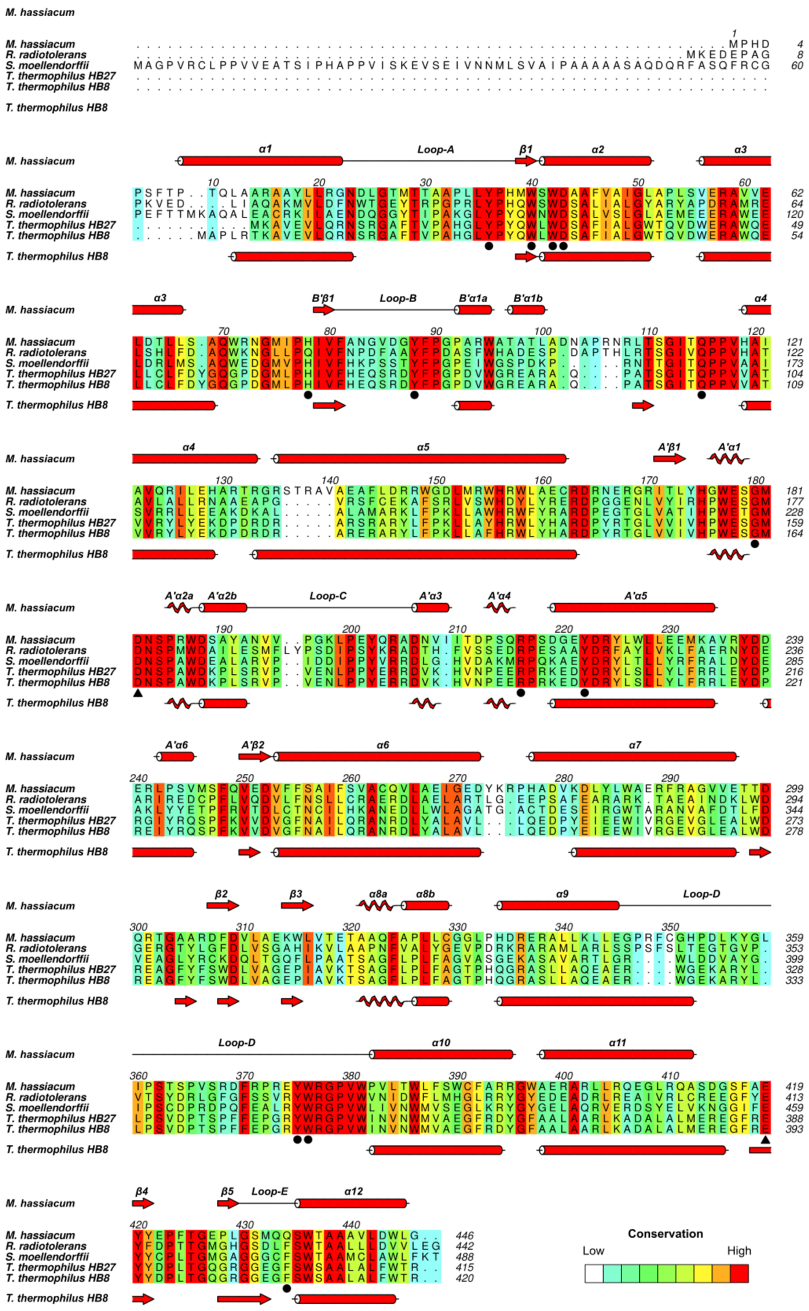

Figure S2 Amino-acid sequence alignment of characterized mannosyl(glycosyl)glycerate hydrolases. Amino-acid sequences of *Mycolicibacterium hassiacum* GgH (GenBank: EKF25940.1) and of MgH enzymes from *Rubrobacter radiotolerans* (GenBank: AFC76324.1), *Selaginella moellendorffii* (GenBank: XP_002961898.1), *Thermus thermophilus* HB27 (GenBank: WP_011173059.1) and *T. thermophilus* HB8 (GenBank: WP_011228344.1) were aligned with *Clustal Omega* (Sievers *et al.*, 2011). Secondary structure elements of *M. hassiacum* GgH and *T. thermophilus* HB8 MgH were identified with *DSSP* (Kabsch & Sander, 1983) and are indicated above and below the alignment, respectively. Residues are coloured from white to red according to increasing conservation. Catalytic residues (*M. hassiacum* Asp182 and Glu419) are marked with black triangles and other substrate-interacting residues with black circles. Figure prepared with *ALINE* (Bond & Schuttelkopf, 2009).

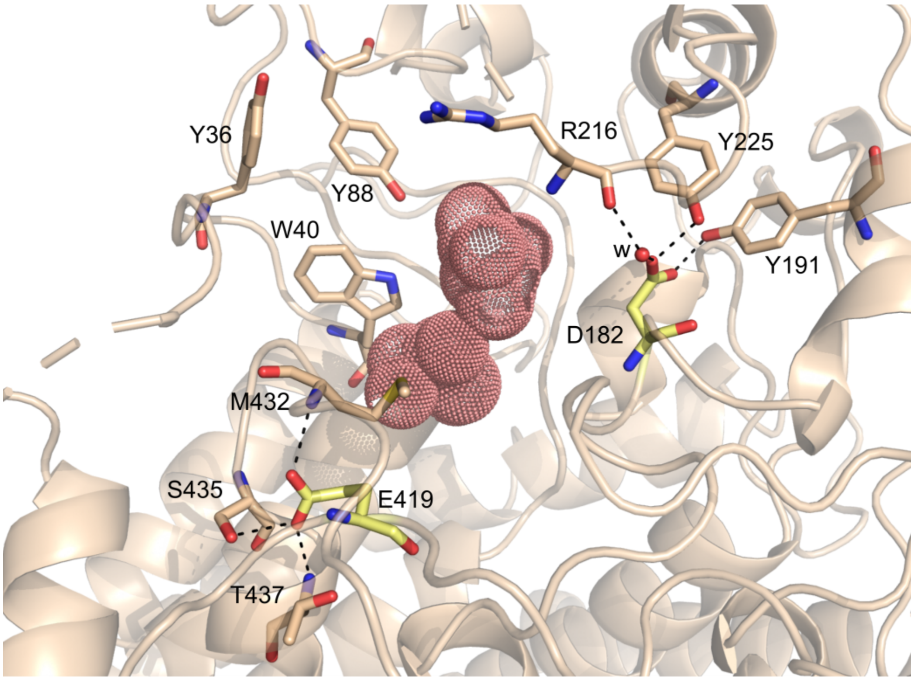

Figure S3 Active-site region of *Mh*GgH in the open state. Catalytic residues (yellow sticks) are pointing away from the active-site cavity (salmon spheres), stabilized by direct hydrogen bonds and by water (w)-mediated contacts with the neighbour residues (dashed lines).

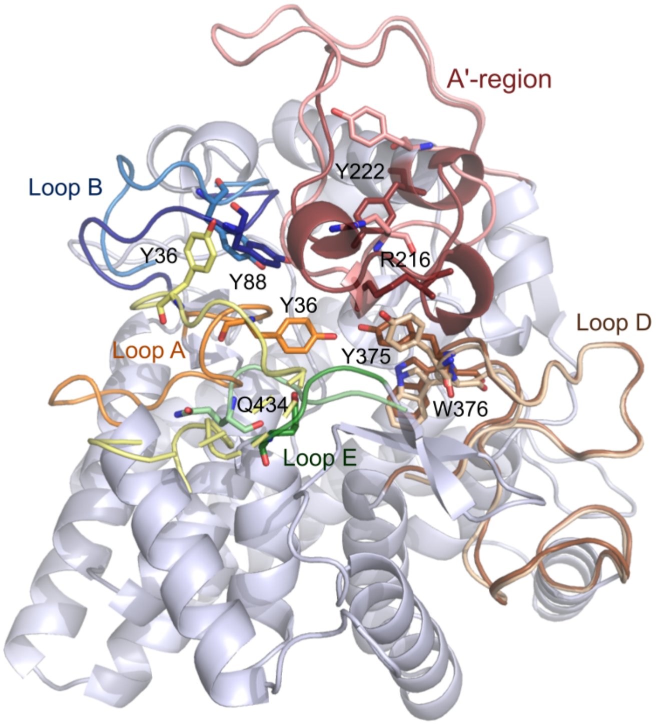

Figure S4 Conformational changes in *Mh*GgH induced by substrate binding. Open (lighter hues) and closed (darker hues) states of monomeric *Mh*GgH are shown. The A'-region (flexible segment), and loops A, B, D and E are coloured salmon, yellow, blue, brown and green, respectively. Some of the substrate-interacting residues present in the highlighted regions [Tyr36 (loop A), Tyr88 (loop B), Arg216, Tyr222 (A'-region), Tyr375, Trp376 (loop D) and Gln434 (loop E)] are represented as sticks.

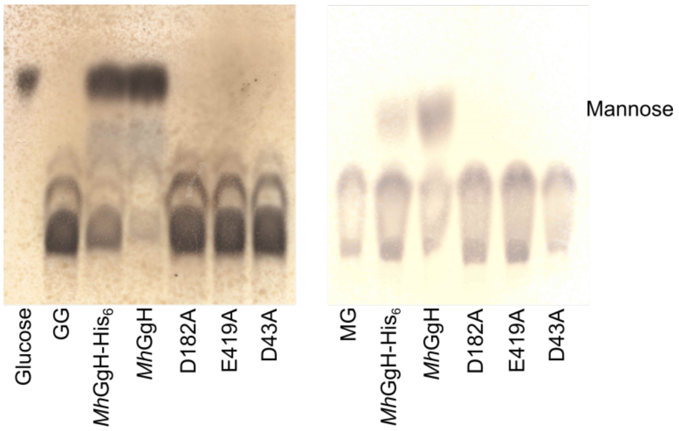

(*a*) (*b*)

Figure S5 Hydrolysis of GG and MG by *Mh*GgH variants. The tagged (*Mh*GgH-His$_6$) and tag-less *Mh*GgH were incubated with GG (*a*) and MG (*b*) and their ability to hydrolyse the compounds was evaluated by TLC. Both tagged and tag-less *Mh*GgH variants were able to hydrolyse GG and MG, however a lower residual substrate amount was detected for the tag-less variant. None of the variants (D182A, E419A and D43A) produced a detectable amount of glucose or mannose.

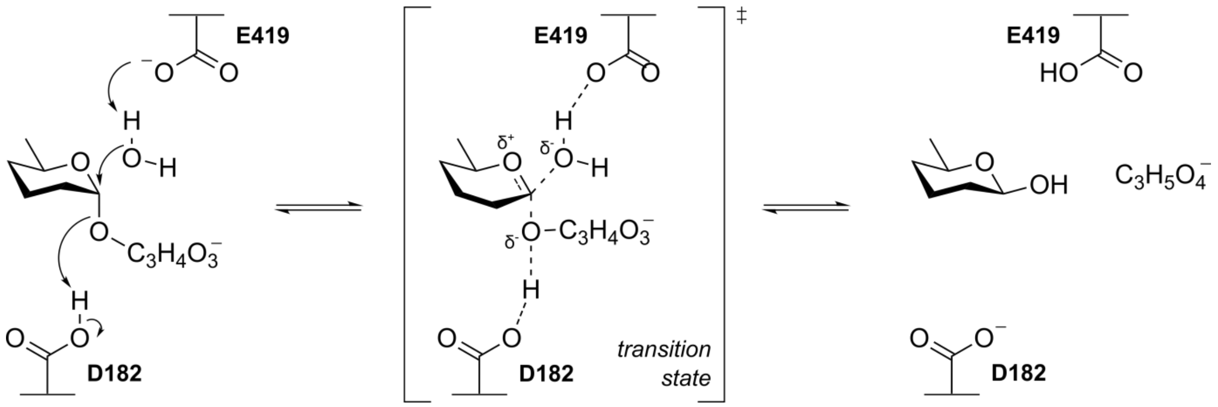

Figure S6 Proposed hydrolysis mechanism of *Mh*GgH. The hydrolysis of GG and MG by *Mh*GgH is proposed to be performed by a classical inverting mechanism, in a single step, involving the catalytic residues Asp182 and Glu419 and the formation of a oxocarbenium ion-like transition state (adapted from Withers, S. and Williams, S. "Glycoside Hydrolases" in CAZypedia, available at URL http://www.cazypedia.org/, accessed 15 April 2019).

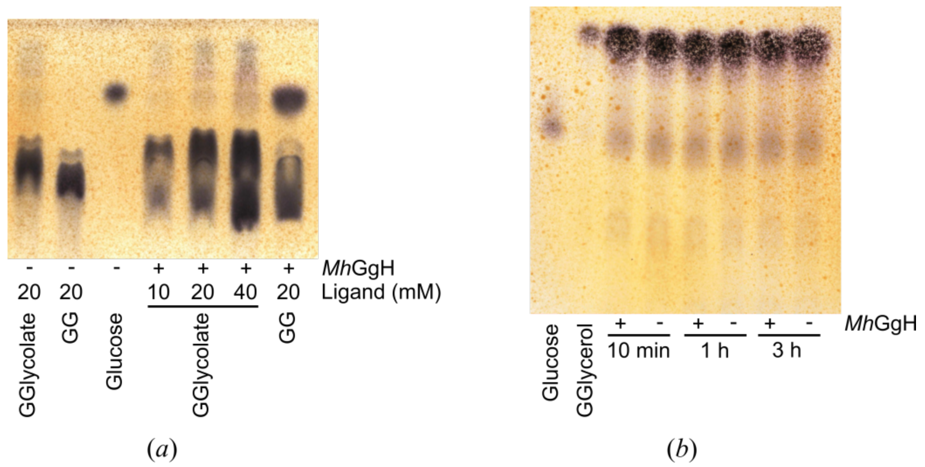

Figure S7 Hydrolysis of GGlycolate and GGlycerol by *Mh*GgH. The ability of *Mh*GgH to hydrolyse GGlycolate (*a*) and GGlycerol (*b*) was evaluated by TLC. (*a*) Different concentrations of GGlycolate were incubated with *Mh*GgH at 50°C for 2h. Glucose production was not detected for any of the concentrations tested. (*b*) *Mh*GgH was incubated with GGlycerol for 10 min, 1h and 3h at 50°C. Production of glucose could not be observed.
